# The effect of metal artefact on the design of custom 3D printed acetabular implants

**DOI:** 10.1186/s41205-020-00074-5

**Published:** 2020-08-26

**Authors:** Anna Di Laura, Johann Henckel, Robert Wescott, Harry Hothi, Alister J. Hart

**Affiliations:** 1 The Royal National Orthopaedic Hospital, Brockley Hill, Stanmore, London, HA7 4LP UK; 2 Institute of Orthopaedics and Musculoskeletal Science, University College London, London, UK

**Keywords:** Paprosky acetabular classification, Revision hip surgery, Metal artefact, Computed tomography, Custom 3D printed implants

## Abstract

**Background:**

3D Printed custom-made implants constitute a viable option in patients with acetabular Paprosky III defects. In these patients, needing complex hip revision surgery, the appreciation of the bony defect is crucial to assure stable fixation of the customised implant, often intended to replace a failed one. We aimed to understand the effect of metal artefact on the design of customised implants.

**Methods:**

26 patients with massive acetabular defects were referred, between May 2016 and September 2018, to our institution classified as “un-reconstructable” by other hospitals. They all received custom 3D-printed acetabular cups. A subset of them underwent two-stage revision surgery due to infection. We then extended the two-stage procedure to the cases where metal artefacts were significantly affecting the reading of the CT scans.

CT scans of patients’ pelvises were taken pre and post-implant removal. We assessed for changes in bony shape and volume of the pelvis using 3D imaging software and quantified the effect on implant design with CAD software.

**Results:**

Eight (out of 26) patients (31%) underwent two-stage revision surgery. The CT bony reconstructions between the two timepoints changed in all cases. The changes were mostly associated to the shape and distribution of the acetabular defects. Three of these cases (37.5%) showed a remarkable difference in the remaining bone that led to a change in implant design. So far, there has been no difference in the clinical outcome between the patients who underwent single (*n* = 18) and two-stage surgery (*n* = 8).

**Conclusions:**

The shape of the acetabulum reconstructed from CT data is potentially altered by metal artefact and bone excised during removal of the failed component. For “end-of-road” acetabular reconstruction, we recommend surgeons consider the use of two-stage surgery to enable a reliable fitting of the complex shape of 3D-printed implants.

## Background

The management of large acetabular defects in hip arthroplasty revision surgery is challenging due to the diversity in remaining pelvic bone stock and quality. Severe bony loss, as a result of a broad spectrum of pathologies, can compromise the fixation and mechanical stability of the implant [1–6] with catastrophic effects.

Classification systems have been designed to define the extent of the remaining bone [7, 8]. Paprosky IIIB defects are the most severe and are characterized by supporting bone loss greater than 60% and significant superior-medial migration of the hip centre of rotation (CoR) [9, 10]. According to the Danish Joint Registry, the prevalence of Paprosky II and III defects and pelvic discontinuity problems are increasing both in absolute numbers and as proportions of total acetabular revision burden. The Norwegian Joint Registry reports 23–30 Paprosky 3B defects per year over the last 5 years. According to the manufacturers, over the last 9 years there has been an exponential increase in the use of custom three-dimensionally 3D-printed implants [11]. An increasing number of primary total hip arthroplasties (THAs) are being performed in younger patients and combined with an aging population, this may explain the growing incidence of revision surgeries, as more patients outlive their prosthesis [12].

Traditionally antiprotrusio cages, spanning the ischium and ilium, have been the preferred devices used for the management of Paprosky IIIB defects [13]. However, their use is associated with high (29%) rates of failure [14]. There is no consensus regarding the best option for reconstructing acetabular defects classified as Paprosky IIIB, or greater.

Patterns of bone deficiency vary depending on the patient’s clinical history. Advances in computed tomography (CT) and additive manufacturing (AM) technology have made it possible to design and manufacture custom titanium acetabular implants to reconstruct such bony defects [13, 15–20].

Reconstructive surgery relies on accurate pre-operative planning [21, 22] and is dependent on the quality of the images acquired. Metal artefact can obscure the true dimension and shape of the bones, making planning and execution of the surgery, as well as designing of the customised components, extremely challenging.

At our Unit, we noticed that in cases of infection requiring two-stage surgery, the post-implant removal scan was more realistic to the bony anatomy present at the second-stage surgery.

We aimed to better understand the effect of metal artefact on the design of customised implants. Our primary objective was to assess the difference in the bony shape and volume pre and post-implant removal; the secondary objective was to assess the design changes, if any, between the two timepoints (pre and post-implant removal).

## Methods

A cohort of 26 patients with massive acetabular defects were referred, between May 2016 and February 2019, to our Institution, classified as “un-reconstructable” by other hospitals (Paprosky IIIB and beyond [8]). The patients received custom 3D printed acetabular components, ProMade™ Lima^†^.

All patients were imaged with a Siemens SOMATOM® Definition AS+ 128 slice CT scanner. Images were acquired at 100 kV, 100 mAs [23]. The effective dose to the patients was 1.4 mSv. Data was saved as DICOM files, anonymised and provided to the manufacturer, via a secure dedicated portal, to be included into a specific workflow for implant design. The patient-specific implant proposal was, in all cases, discussed between the surgeon and the engineers to optimise the surgical procedure. All surgeries were performed by a single consultant orthopaedic surgeon.

At first, the use of two-stage surgery was evaluated in cases of infection. We then extended the procedure to the cases where metal artefact was significantly affecting the reading of the CT scans and where poor fitting of the new implant would have had a catastrophic effect to the patient.

Eligibility criteria were based on: 1) the clinical history; 2) the quality of pre-op CT scans based on presence of metal artefact and; 3) the severity and complexity of the acetabular defect (Paprosky IIIB and beyond). For the patients undergoing two-stage reconstructive surgery, two Metal Artefact Reduction Sequence (MARS) CT scans were taken, prior and after (the interval CT scan) the removal of the failed prosthesis.

**Fig. 1:**
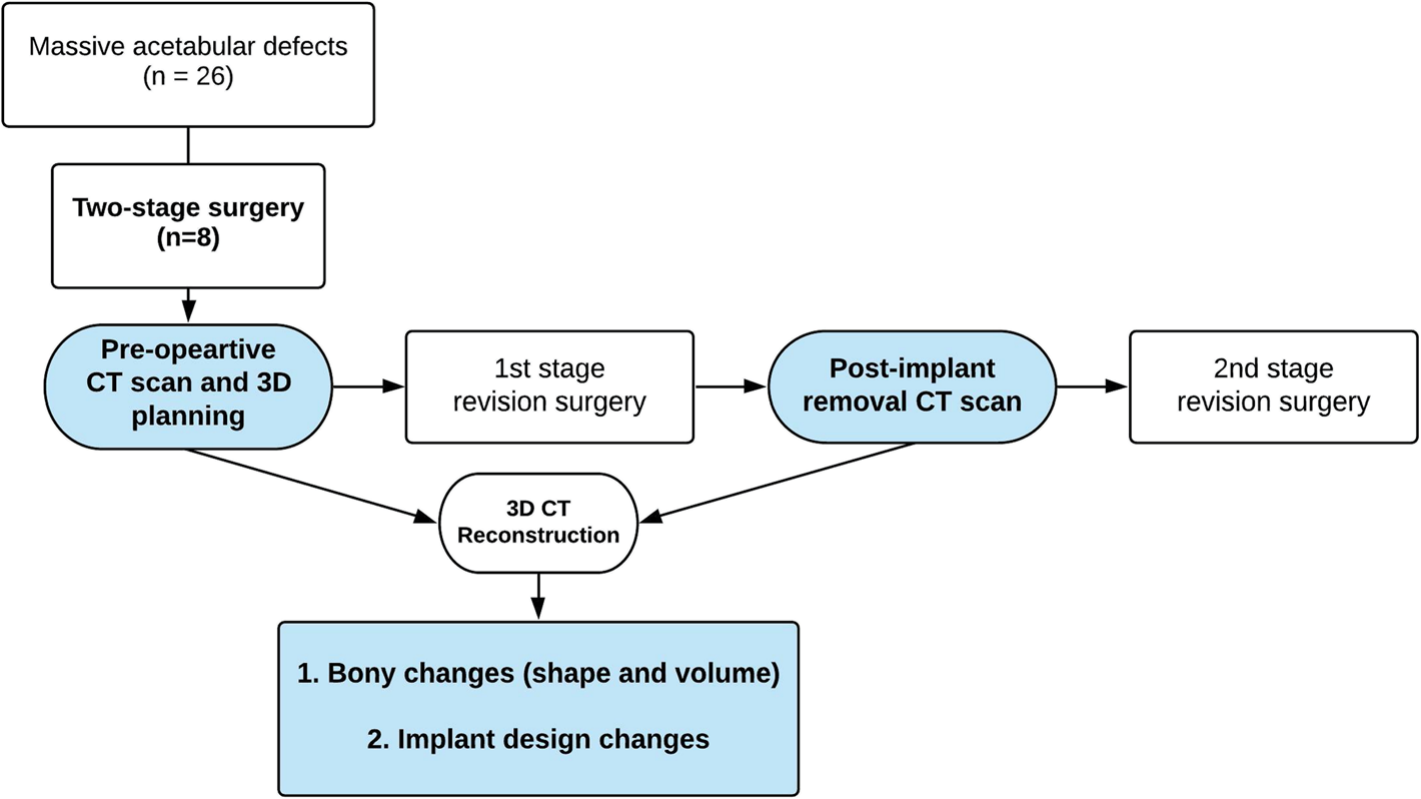
Flowchart of the study design

For the purpose of this study, both scans (pre-operative and interval CTs) were segmented to generate the 3D model of the pelvis and were treated as the scan onto which perform implant design and plan the surgical procedure, Fig. 1.

The outcome measures were:

1. Changes in shape and volume of the innominate bone pre and post-implant removal;
2. Changes in implant design between the two timepoints.

### Prior to implant removal

**Fig. 2:**
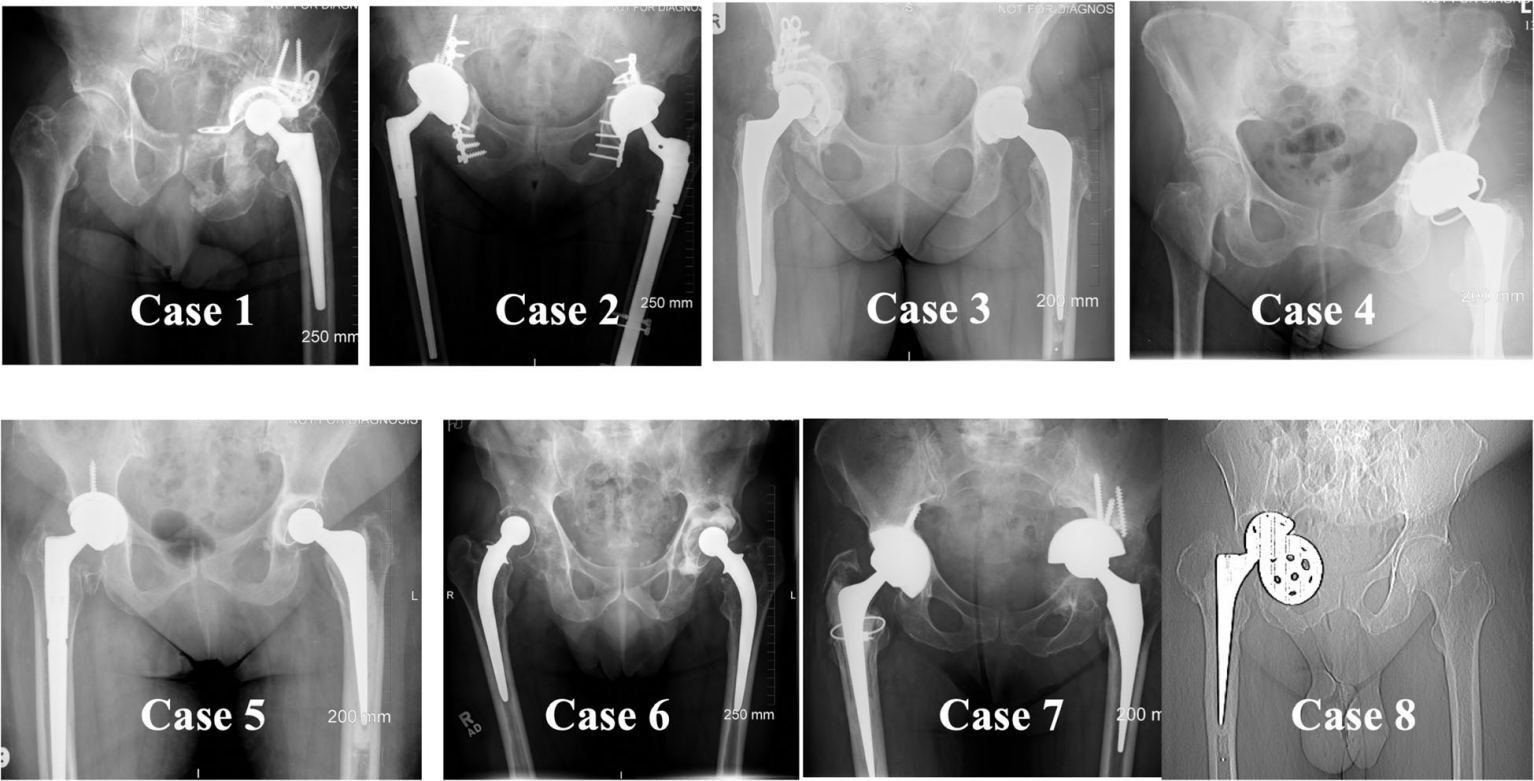
Pre-operative radiographs of the 8 patients included in the study. The acetabular defects were classified as Paprosky IIIB and beyond, there were three pelvic discontinuities (cases 2, 7 and 8)

Acetabular defects were initially classified according to the Paprosky classification system based on the preoperative anteroposterior (AP) pelvic radiograph by the senior consultant orthopaedic surgeon, Fig. 2.

3D reconstruction of the patients’ bony pelvises were generated by segmentation via commercially available software (Mimics 19.0, Materialise, Belgium). Data from CT scans was used for accurate assessment of the centre of rotation of the failed hip. Integrity of the acetabular rim and anterior and posterior columns were assessed. The volume of the reconstructed innominate bone (defect side) was measured and recorded to be compared to the reconstructed bone after the removal of the failed implant/component.

### The first stage procedure

All surgeries were performed using an extensive posterior approach. The failed components were removed. In two cases, a spacer was inserted into the acetabular cavity to treat the infection (Case 2 and 3).

### Post implant removal

**Fig. 3:**
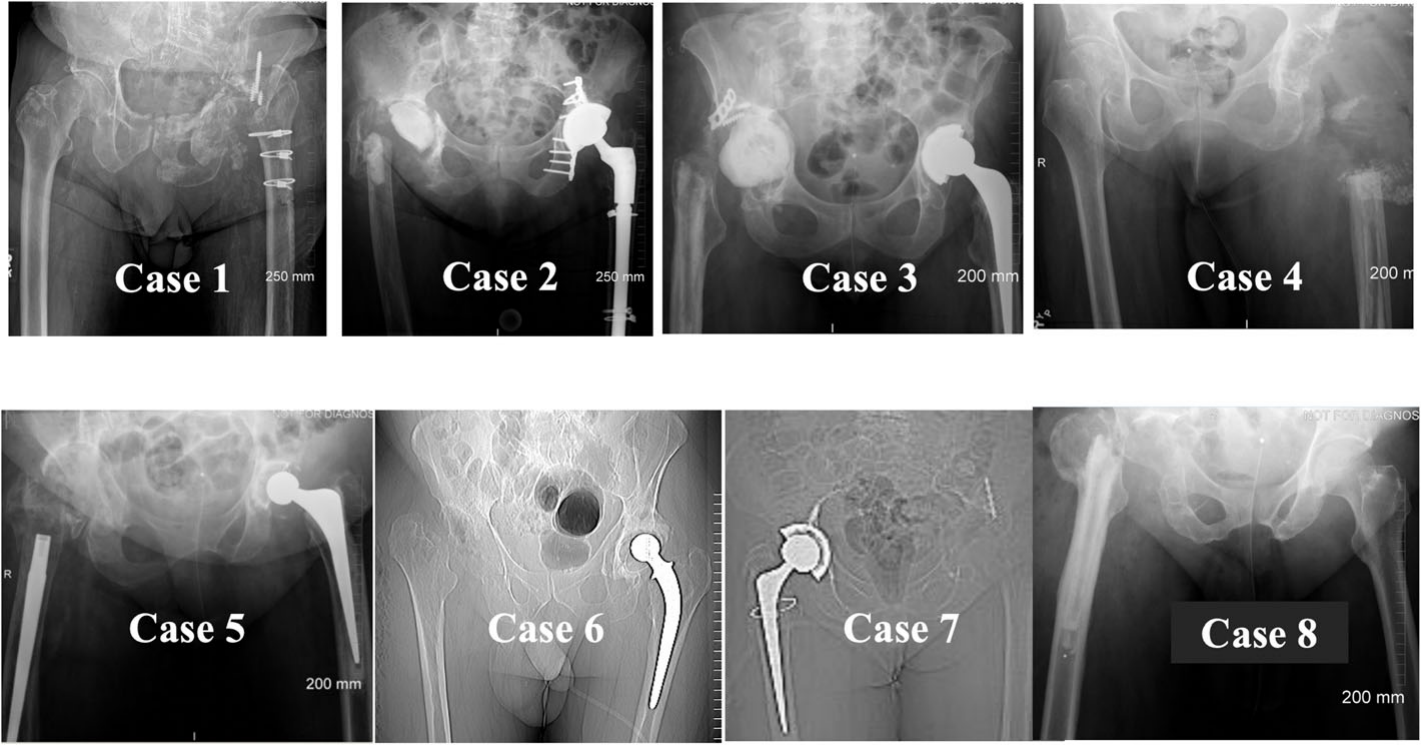
Series of radiographs taken after the removal of the implants showing the remaining bone stock. In Cases 2 and 3, the acetabular spacer can be seen

Following removal of the implant, the patients underwent radiography and CT scanning of their pelvises, Fig. 3.

**Fig. 4:**
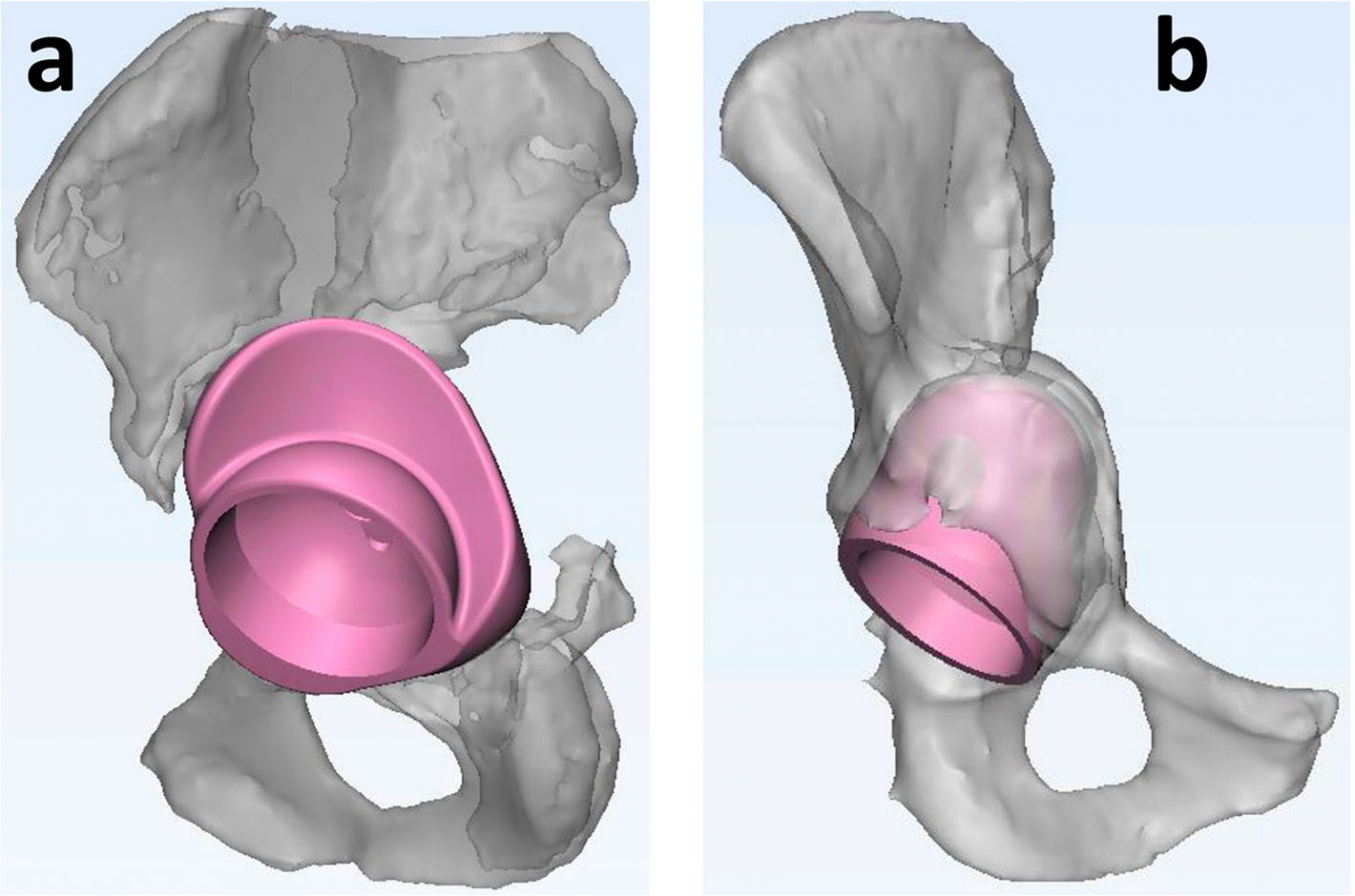
Explanatory example (lateral **a** and anterior-posterior **b** views) of the “patient-specific augment” technique used to estimate defect size

The acetabular bone loss, or defect size, was estimated on an implant-based analysis consisting of filling the defect with the future implant, defined as “patient-specific augment”, aiming at restoring the biomechanics of the joint. For each case, a defect filling volume was designed, starting from the bony shape, Fig. 4, and the volume was measured.

The difference in bony size (innominate bone) between the pre-operative CT-based defect assessment and measurements made on the post-operative CT scan were compared. The segmented 3D models were exported to STereoLithography (STL) files and the volumes co-registered to compute the difference between the two reconstructions. Bone shape changes, or residual STL volume, refers to the difference between the files generated at two timepoints. It is intended as the subtraction of the intersection of the two volumes from their union [24]. Segmentation of CT scans was performed by experienced engineers to minimise errors in the reconstruction of the anatomy, due to a reliance on user selection of bony landmarks. Segmentation time and any difficulties encountered were also recorded.

### Designing of the implant

**Fig. 5:**
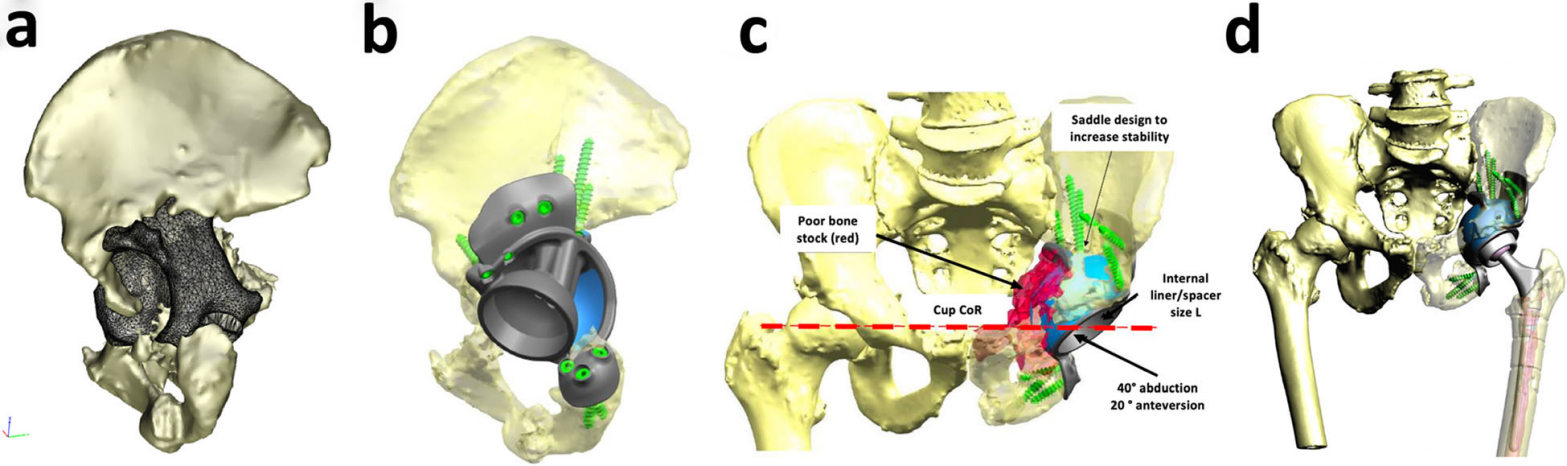
Pre-operative planning workflow. The acetabular bone loss, or defect size, was estimated on an implant-based analysis consisting of filling the defect with the future implant (**a** and **b**), the implant geometry, flanges and screw holes were designed to enhance implant to bone surface area (**b**). The selection of component types aimed at restoring joint biomechanics, centre of rotation and offsets (**c** and **d**)

Designing the custom titanium implant involved the following key steps: 1) filling the defect with porous titanium, 2) assuring fixation with structural titanium and screw holes and 3) determining the optimal location of centre of rotation, Fig. 5. Once approved, the implants were produced using EBM additive manufacturing with regions of trabecular titanium to promote osteointegration [25, 26]. Alongside the titanium implant, plastic models of the patient’s pelvis, the custom implant and the drill guides were manufactured using 3D printing and sterilised for intraoperative use. Component design and pre-operative planning was undertaken with close collaboration between surgeon and engineer.

### The second stage procedure

The hip joint was accessed via a posterior approach often using the existing scar. The dissection through the gluteal muscles depended on the exposure required. With the acetabulum exposed and the existing component explanted, the bone was prepared with removal of osteophytes for the custom implant. The surgeon used 3D printed plastic pelvic and implant models to better appreciate the size of the defect, to assist with surgical exposure, to guide surgical orientation and to prepare the bone.

### Post-operative radiological assessment

All patients underwent conventional standing anteroposterior (AP) view radiographs of the pelvis and the hip post-operatively. Post-operative evaluation of reconstruction was achieved by evaluation of AP X-rays and standing bi-planar X-rays system (EOS Imaging, Paris, France) from the Picture Archiving and Communications Systems (PACS). The surgeon assessed implant position as well as the restoration of centre of rotation. Complications within the first 6 weeks of surgery were recorded.

## Statistics

Statistical analysis was performed using Prism 7 (GraphPad Software, San Diego, CA, USA). *T-test* was used to determine if there was a difference in volume between hemipelvises generated before and after the removal of the implant, as well as if there were differences in segmentation time between the two scans. The level of significance for all statistical analyses was *p* < 0.05.

## Results

Eight patients (8/26) underwent a two-stage revision surgery to allow the design of the custom-made titanium acetabular component, 4 due to infection and 4 due to significant metal artefacts. Time between post-implant removal CT scan and second-stage surgery ranged from 1 to 5 months. The mean age of patients was 75 years (range 62–88) and mean weight was 76 Kg (range 60–98). Patients’ demographics and clinical data are reported in Table 1.

**Table 1:** Patients’ characteristics

Case	Gender	Age, Years	Weight, [Kg]	Reason for Revision	Paprosky Classification	Time between Surgeries
1	M	82	70	Infection (L)	IIIB	29 months
2	F	71	70	Infection and pelvic discontinuity (R)	Discontinuity	16 weeks
3	F	70	68	Loosening and infection (R)	IIIB	13 weeks
4	F	79	82	Recurrent dislocation and infection (L)	IIIB	19 weeks
5	F	88	60	Loose R hip replacement with inflammatory pseudotumor	IIIB	5 weeks
6	M	62	85	Loose R acetabulum MoP replacement with subluxation	IIIB	5 weeks
7	F	74	76	Loose L cup migrated medially into the pelvis	Discontinuity	7 weeks
8	M	70	98	Acetabular cup failure following primary R THA	Discontinuity	12 weeks

*R* Right hip, *L* Left hip, *MoP*, Metal-on-Polyethylene bearing type

### Bone shape changes

In virtually all cases, the bony shape changed between the initial CT (with implant) and that taken following the first operation (without implant).

We found that areas clearly defined on interval CT reconstructed models were missing on the pre-operative CT reconstructions, and the acetabular rim definition was not clear on the 3D reconstructions derived from pre implant removal scans.

**Fig. 6:**
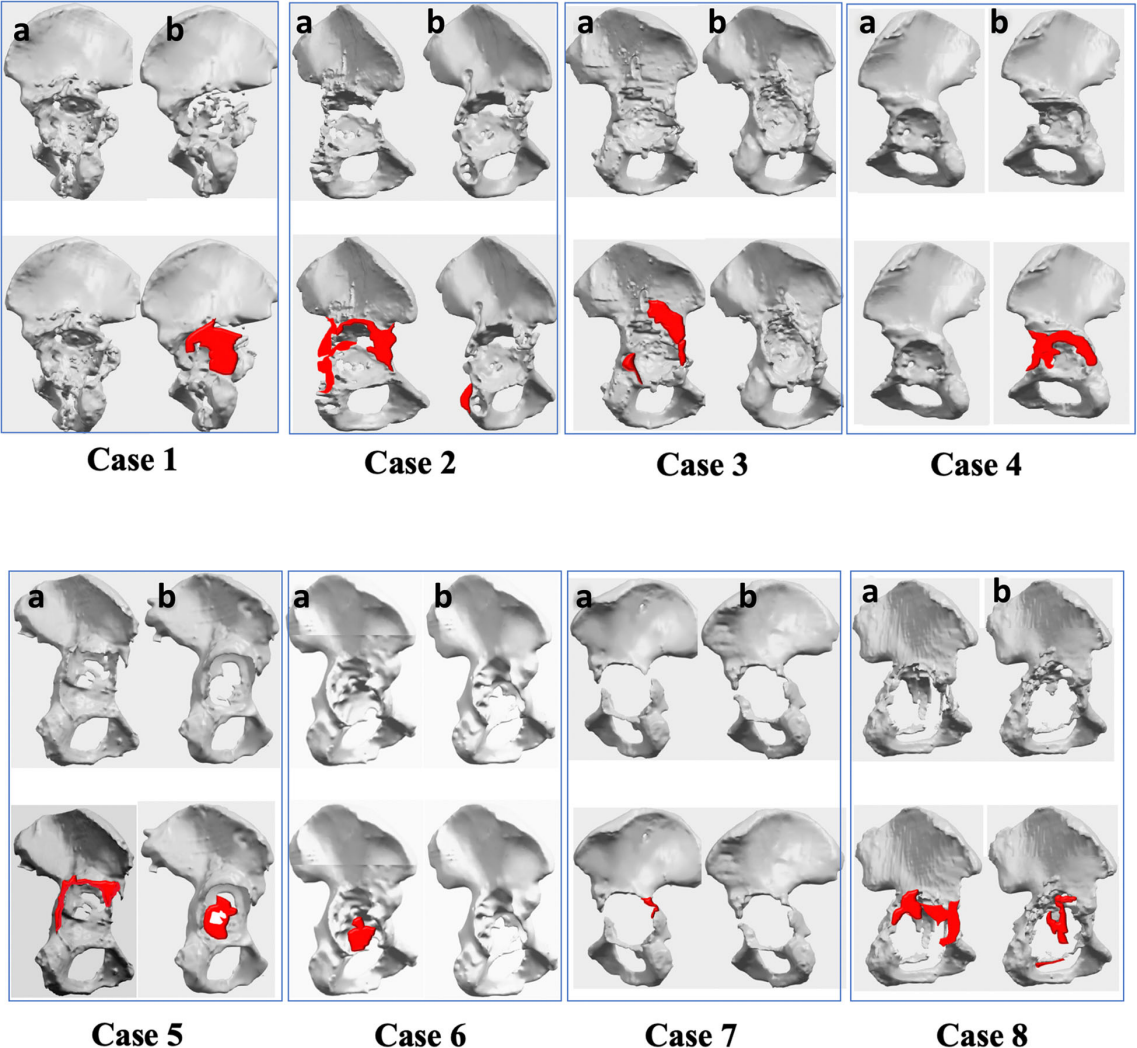
Pre and post implant removal reconstructed anatomies, **a** and **b** respectively. Parts in red represent the missing bone between the 2 CT reconstructions . Case 1: The volume of metal adjacent to the superior rim (cage, screws and femoral head) dramatically affected the true bony anatomy of the superior rim which resulted in a major change in the design of the 3D printed implant. Cases 2, 3, 5 and 6: images **a** showed reduced bone in the superior acetabular rim, **b** showed absent medial acetabular wall due to bone removed at surgery or not clearly visible on pre-removal scan. Case 4: the anterior wall was absent in the post-implant removal CT reconstruction (**b**). For case 7 and 8, the acetabular implant was loose and migrated into the pelvis and away from the innominate bone. Minimal differences between pre and post implant removal scans were seen

**Fig. 7:**
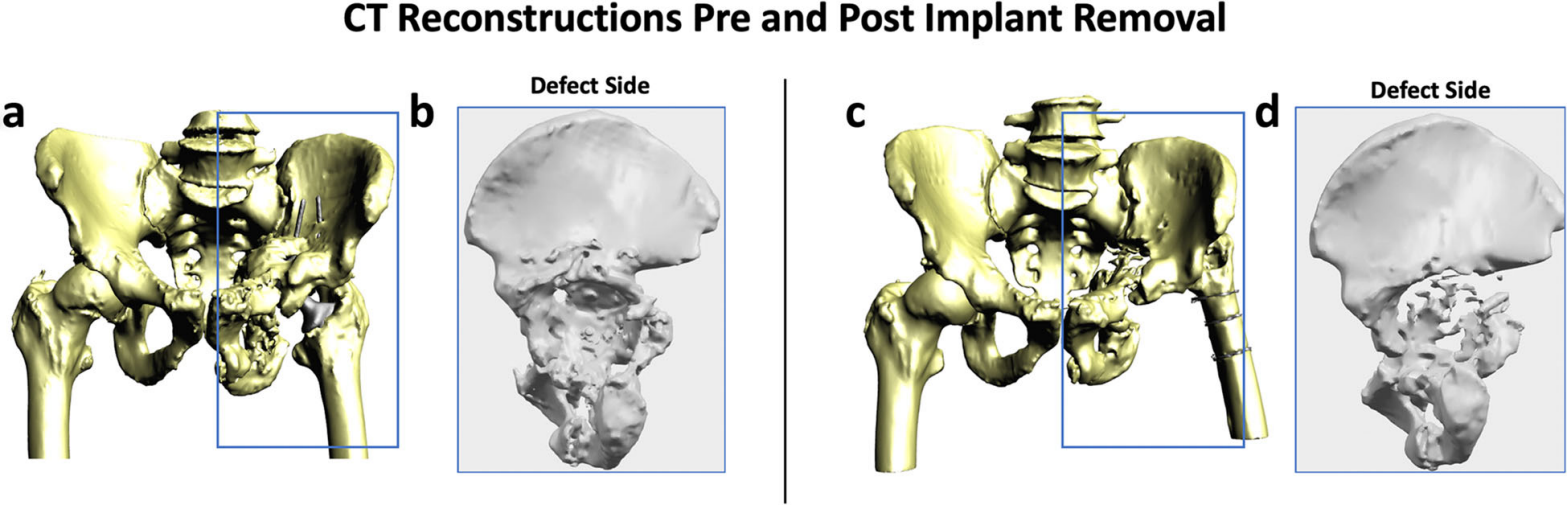
Reconstructed pelvic models of Case 1 both pre and post implant removal. Image **a** shows the reconstructed pelvis and femurs prior to the removal of the implant; image **b** shows details of the acetabular cavity at the defect side with considerable amount of bone loss (lateral view). Image **c** shows the reconstructed pelvis and femurs after the removal of the implant; image **d** shows details of the acetabular cavity with numerous bone fragments at medial acetabular wall (lateral view)

The cases and patterns of bony shape pre and post-implant removal are summarised in Fig. 6. In detail: Case 1: The volume of metal adjacent to the superior rim (cage, screws and femoral head) dramatically affected the true bony anatomy of the superior rim which resulted in a major change in the design of the 3D printed implant (see Fig. 7). Case 4: There was a major soft tissue defect with dead proximal femoral bone requiring proximal femoral replacement. To maximise the fixation of implants to the pelvis in such a hostile environment, the surgeon opted for a custom implant so that the constrained liner used at revision surgery did not pull the implant off the bone. In this case two-stage surgery was used to maximise the chance of limb salvage.Case 5: Image A shows reduced bone in the superior acetabular rim. B shows absent medial acetabular wall due to bone removed at surgery or not clearly visible on pre-removal scan. This pattern was observed in cases 2, 3 and 6 as well. Case 7: The acetabular implant was loose and migrated into the pelvis and away from the innominate bone and therefore there were minimal differences between pre and post implant removal scans. This was seen in case 8 as well, where the loose implant was pushed into the pelvis, hence away from the pelvic bone.

### Bone volume changes

The absence of metal artefacts in the post-operative CT scans reduced the uncertainty in areas where artefacts misled the anatomical reconstructions on pre-operative CT scans, hiding bony regions or preventing a clear bone boundary from being discerned.

The volume of the innominate bone (defect side) on pre-implant removal CT reconstructed virtual models was a mean (±SD) of 269 (± 65.40) cm^3^ (min = 192, max = 367 cm^3^).

The volume of the innominate bone (defect side) on post-implant removal CT reconstructed virtual models was a mean (±SD) of 264 (±48.34) cm^3^ (min = 209, max = 337 cm^3^).

The difference was not statistically significant (*p* = 0.66, paired t-test).

The defect size, or “augment size” was a median of 77 cm^3^ (range 33–95 cm^3^) representing, on average the 27% of the innominate.

An estimation of the volume of bone obscured by metal artefacts in the pre-operative CT scans was a median of 10 cm^3^; an estimation of the bone removed at surgery in the post-operative CT scans was a median of 14 cm^3^.

Segmentation time was two-fold longer when the prosthesis was in-situ, mean (±SD), 2.7 (±1) Vs 5.5 (±2) respectively.

### Design changes

**Fig. 8:**
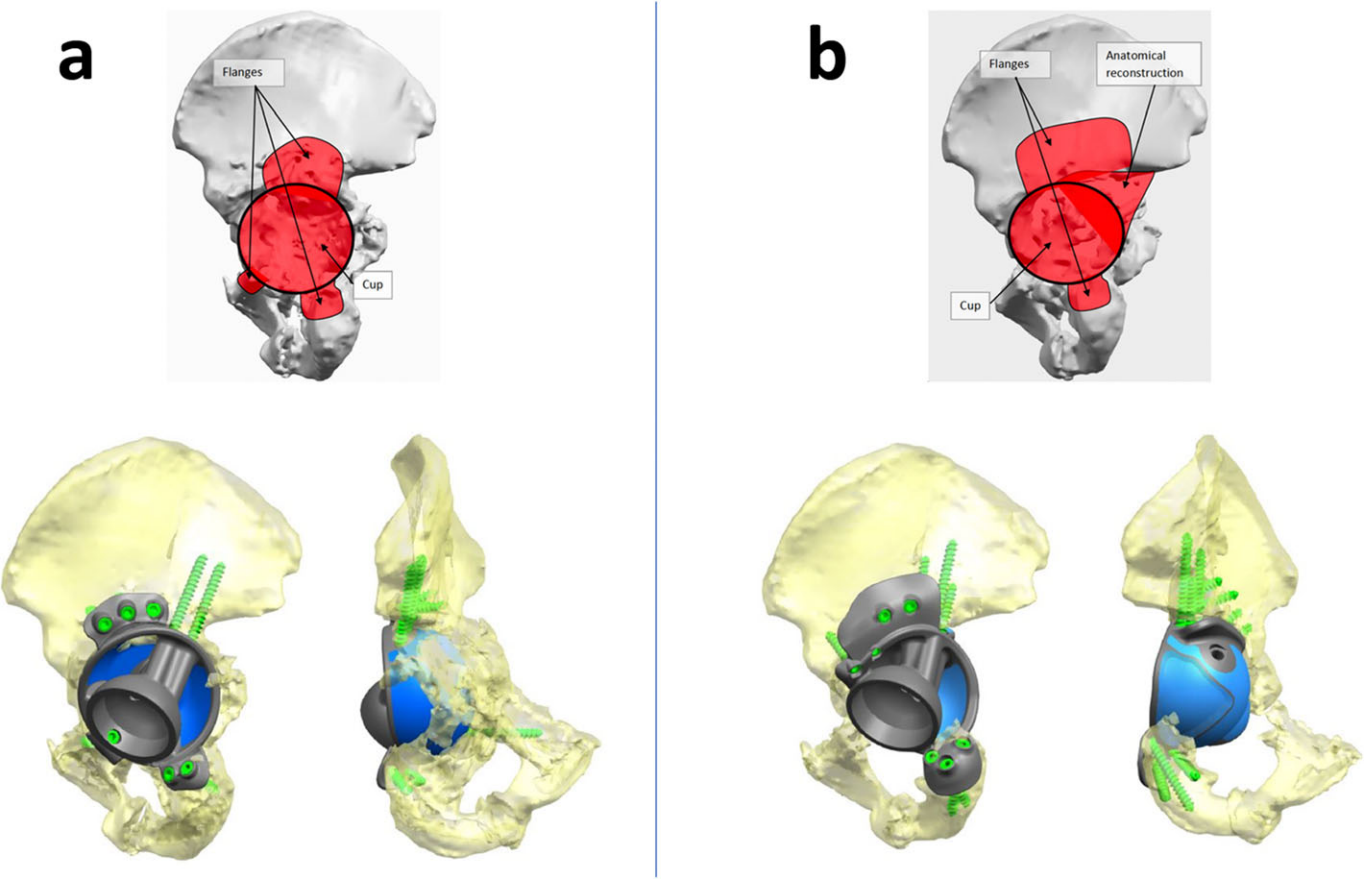
Case example. 3D bony reconstructions of the hemipelvis (defect side) of Case 1 with relative implant design (**a**) prior to and (**b**) following 1st-stage surgery. Differences in the remaining bone at the medial acetabular wall and rim can be seen - these led to a change in the number and shape of implant flanges and number and location of screws

Three of the cases (37,5%; Cases 1, 4 and 5) showed a remarkable difference in the shape of the remaining bone that led to a change in implant design (Fig. 8**).**

The remaning cases resulted in minor changes of the custom implant design and/or changes in the method of preparation of the bone.

### Post-operative radiological assessment

- Two-stage revision patients (*n* = 8)

**Fig. 9:**
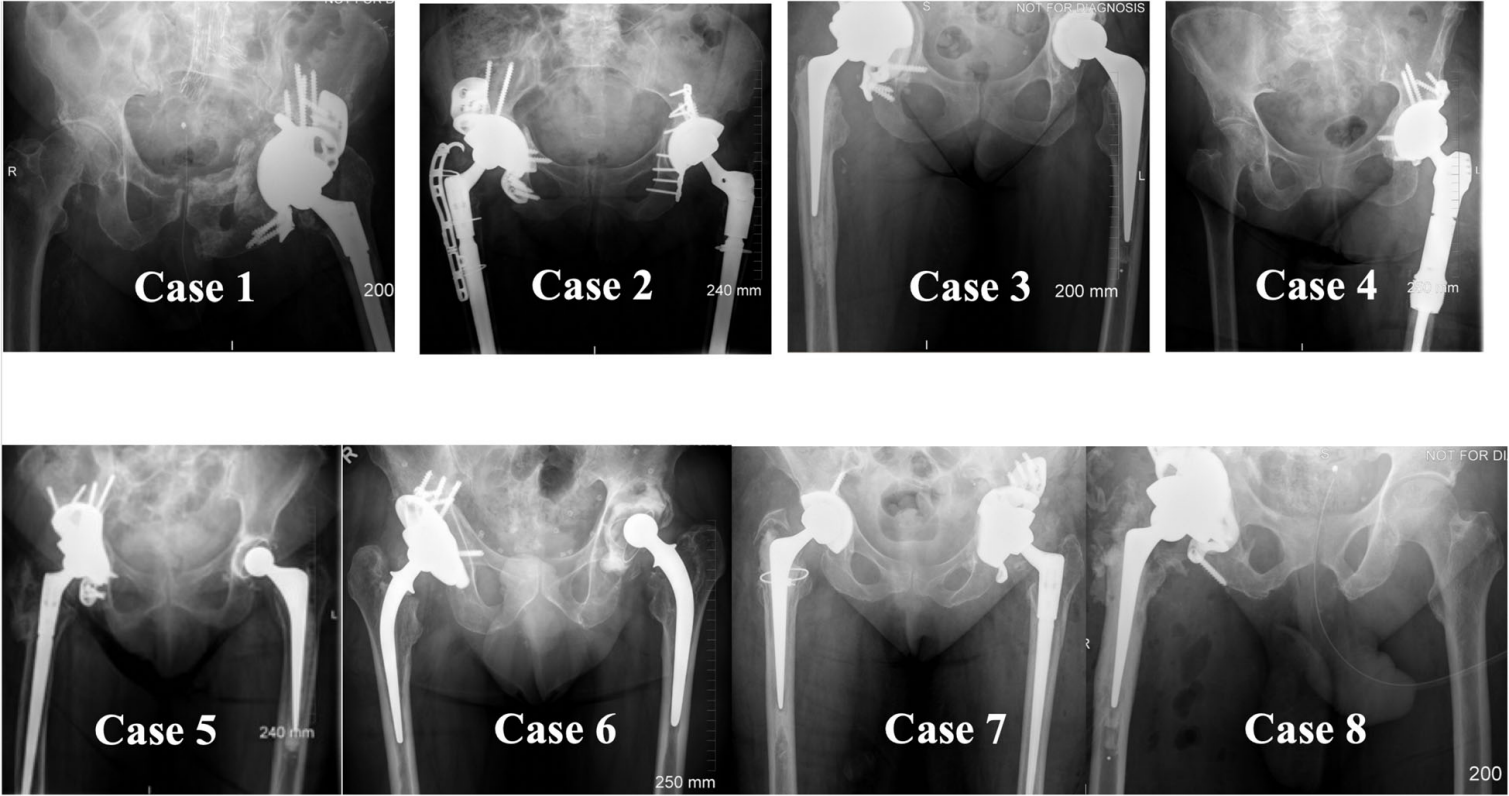
Post-operative radiographs of the 8 patients with the custom metal work in place

Follow-up time ranged between 15 and 32 months (median = 20.5 months), and the post-operative course was uneventful for the 8 patients. The components were congruent, no case showed evidence of early loosening. Two dislocations occurred post-op and were reduced by closed reduction and none of the patients have had revision for dislocation at the time of publication. Mean oxford hip score at latest follow up was 30.3. Standard 2D imaging showed satisfactory restoration of centre of rotation, Fig. 9.

- Single-stage revision patients (*n* = 18)

Follow-up time ranged between 15 and 45 months (median 30.5 months). Mean oxford hip score at latest follow up was 30.6. To date one complication has occurred, a comminuted fracture of the iliac crest. The component was considered stable at placement and no fracture was suspected intraoperatively. In addition, no fracture was apparent on immediate postoperative imaging (plain radiographs). Early ambulation occurred postoperatively, during which the patient suspected that the component had migrated. Subsequent imaging (CT and plain radiograph) revealed the fracture. The patient underwent surgery for removal of the implant, no further surgery was performed. None of the patients has had revision surgery at the time of publication. Radiologically, the components were stable, no signs of loosening were observed.

## Discussion

This study investigates the use of two-stage procedures for the reconstruction of massive acetabular defects, in case and in absence of infection. Our aim was to understand to what extent bony information obscured due to metal artefacts can affect the design of customised implants.

We compared 3D-CT scans taken of patients pre and post-implant removal and found that the bone shape changed in all cases. The medial acetabular wall was often missing from the interval 3D reconstruction (post implant removal) and acetabular rim definition was not clear on the 3D reconstructions derived from pre-implant-removal scans. Also, bone is inevitably removed during initial implant removal. These concomitant variables led, in some cases, to design modifications.

Two main problems arise when treating patients with massive acetabular defects: (1) Poor visualisation of the acetabular defect and construct on computed tomography (CT) due to the presence of metal-related beam hardening artifacts and (2) inevitable bone loss associated with removal of the failed implant at surgery, changing the architecture of the defect. This “new” intra-operative defect cannot be predicted on the pre-op CT.

The adoption of the two-stage procedure began with the treatment of patients for infection in the presence of a massive acetabular defect. Application of this technique was then expanded to cases where large amounts of metal work obscured the bone, making the designing greatly uncertain. Finally, we extended the indication to “end-of-road” reconstruction to do everything possible to increase the certainty of good fixation of the customised implant to the pelvis.

Comparison of pre and post-implant removal scans allowed us to estimate bone that was obscured by metal artefact or subsequently removed intraoperatively.

We observed that the virtual 3D-CT bony models changed in all cases. A prevalence of areas of the medial acetabular wall missing from the interval 3D reconstruction (post implant removal) was noted, whereas acetabular rim definition was not clear on the 3D reconstructions derived from pre-implant removal scans.

Although the overall innominate bone volumes did not change significantly between the two timepoints, the changes were predominately associated with the shape and distribution of the acetabular defects (Figs. 6 and 8). This is of clinical significance as it leads to implant design modifications and may ultimately change the surgical procedure and instruments needed. If not anticipated, these changes can presumably increase the surgical time and lead to poor implant fitting.

Previous studies have highlighted the difficulty of positioning a custom made implant accurately in patients with a massive acetabular defect [27, 28]. Reports on the clinical outcome in the literature are sparse. One of the main challenges is achieving accurate fitting to the patient’s host bone. Additive manufacturing has enabled the accurate production of bespoke titanium implants with integral porosity providing a fairly new tool to manage massive acetabular defects in revision hip surgery.

CT data is a potential source of error when designing and placing custom-made components in patients with massive acetabular defects [29]. Despite advancements in CT technology, metal artefacts mislead the anatomical reconstruction by hiding bone portions or preventing a clear bone boundary reading. Difficulty following the preoperative plan, and press fitting the custom component, arise when intraoperative defect topography differed from that seen on CT.

There is no patient specific instrumentation or image technology solution to overcome the issue of reconstruction errors due to metal noise or to predict the amount and shape of bone removed at revision.

Two-stage revision THA is the gold standard to treat infection, or periprosthetic joint infection (PJI) [30–34]. The morbidity and mortality of patients undergoing a second-stage revision THA for the treatment of PJI is higher compared to single-stage surgery, linked to the necessity of two major surgical procedures. It has been observed that the main risk factors for a two-stage exchange failure include hemodialysis, obesity, multiple previous procedures, diabetes mellitus, corticosteroid therapy, hypoalbuminemia, blood transfusion, immunosuppression, rheumatological conditions, and coagulation disorders [35]. These factors together with the aim to improve long-term clinical outcomes are always to be consider in case-by-case decision making.

We acknowledge the following limitations. Firstly**,** the two-stage surgical procedure, with pre-operative and interval CT scanning, doubles the radiation dose to the patients. This has constituted, and continues to be, a limiting aspect for the use of computed tomography. However, new CT scanners with optimized sequences have reduced the dose penalty by up to 50% [36]. Secondly, manual segmentation can lead to errors in the reconstruction of the anatomy, due to a reliance on user selection of bony landmarks. However, segmentation of CT scans was performed by experienced engineers. Lastly, we acknowledge the small sample size of these extremely difficult cases, although our hospital constitutes the largest orthopaedic hospital in the UK, highlighting the rarity of their kind.

## Conclusions

Revision arthroplasty in patients with severe acetabular defects is a challenge, even for the most experienced orthopaedic surgeon. An accurate representation of the geometry of the defect during pre-operative planning and implant design is crucial for obtaining stable fixation, restoring biomechanics and ensuring long-term survivorship of the prosthesis. Our findings have shown that (1) metal artefacts in pre-revision diagnostic imaging can hinder the visualisation of underlying bone and that (2) bone excision at revision surgery can alter the defect geometry.

In complex “end-of-road” revision cases, where artefacts mislead the anatomical reconstructions on pre-operative CT scans and when PJI has to be treated, we recommend surgeons consider the use of two-stage surgery. Imaging patients post-implant removal and using these images to design the custom implant enables a reliable fitting of the complex shape of 3D printed implants. Further investigation and longer-term follow-up studies are needed to understand which patients would benefit most from this two-stage procedure.

† Lima Corporate, San Daniele del Friuli, Italy

## Data Availability

The datasets used and/or analysed during the current study are available from the corresponding author on reasonable request.
